# Experimental Study of the Factors Influencing the Regeneration Performance of Reduced Graphite Oxide Filter Materials under Water Cleaning

**DOI:** 10.3390/ma16114033

**Published:** 2023-05-28

**Authors:** Min Yang, Bing Yang, Xin Zhang, Saisai Wu, Tao Yu, Hong Song, Fei Ren, Puchun He, Yanhui Zhu

**Affiliations:** 1 School of Resources Engineering, Xi’an University of Architecture and Technology, Xi’an 710055, Chinaybing@xauat.edu.cn (B.Y.); 2 Wuhan Second Ship Design and Research Institute, Wuhan 430205, China; yut04@163.com; 3 School of Management, Xi’an University of Architecture and Technology, Xi’an 710055, China; songhong@xauat.edu.cn; 4 XAUAT Engineering Technology Co., Ltd., Xi’an 710055, China; jkdgcjs@xauat.edu.cn; 5 Yan’an Branch of Shaanxi Provincial Land Engineering Construction Group Co., Ltd., Yan’an 716000, China; hepuchun123@163.com; 6 Hunan Geological Exploration Institute of China Metallurgical Geology Bureau, Changsha 410001, China; balancezq@163.com

**Keywords:** water cleaning, reduced graphite oxide filter materials, cleanliness, filtration performance, regeneration frequency

## Abstract

With the normalization of epidemic prevention and control, air filters are being used and replaced more frequently. How to efficiently utilize air filter materials and determining whether they have regenerative properties have become current research hotspots. This paper discusses the regeneration performance of reduced graphite oxide filter materials, which were studied in depth using water cleaning and the relevant parameters, including the cleaning times. The results showed that water cleaning was most effective using a 20 L/(s·m^2^) water flow velocity with a 17 s cleaning time. The filtration efficiency decreased as the number of cleanings increased. Compared to the blank group, the filter material’s PM_10_ filtration efficiency decreased by 0.8%, 19.4%, 26.5%, and 32.4% after the first to fourth cleanings, respectively. The filter material’s PM_2.5_ filtration efficiency increased by 12.5% after the first cleaning, and decreased by 12.9%, 17.6%, and 30.2% after the second to fourth cleanings, respectively. The filter material’s PM_1.0_ filtration efficiency increased by 22.7% after the first cleaning, and decreased by 8.1%, 13.8%, and 24.5% after the second to fourth cleanings, respectively. Water cleaning mainly affected the filtration efficiency of particulates sized 0.3–2.5 μm. Reduced graphite oxide air filter materials could be water washed twice and maintain cleanliness equal to 90% of the original filter material. Water washing more than twice could not achieve the standard cleanliness equal to 85% of the original filter material. These data provide useful reference values for the evaluation of the filter materials’ regeneration performance.

## 1. Introduction

Compound air pollution seriously affects people’s normal travel and brings varying degrees of harm to people’s bodies [1,2,3]. The relevant literature indicates that fine particles could easily penetrate human lungs and bronchi, and harmful gases and bacterial microorganisms could cause serious damage to the human immune system [4,5,6]. With the normalization of epidemic prevention and control, more people are increasingly concerned about the health and safety of indoor environments [7]. How to maintain a healthy indoor environment has become a consensus issue among people worldwide.

Air filters have been widely used as an effective method to address indoor pollutants [8]. As air filters are more frequently used on a large scale in the context of normalized epidemic prevention and control, they are also more frequently replaced [9]. This is because the air filters’ resistance increased, their filtration efficiency decreased, or they failed to operate properly due to the adsorption and filtration of many solid particles, gaseous pollutants, and biological aerosols trapped by the fibers’ surfaces and deep layers after a period of use [10,11,12]. Filters can also easily breed bacteria, which might cause secondary indoor pollution [13]. The usual solution at present is to directly replace filters; used filter materials are usually directly discarded or burned, which not only pollutes the environment, but also wastes resources [14,15]. Therefore, how to efficiently utilize air filters, whether they have regenerative performance, and whether the performance indicators after regeneration are stable, have become research hotspots.

Many researchers from different countries have conducted related studies regarding these practical issues [16,17,18,19], focusing on improving the filtration efficiency [16], optimizing the filter structure [17], and developing new materials [18,19]. However, these research results have not effectively solved the problem of dust retention in the filter materials, or the problems caused by the filter fiber materials’ low efficiency and high resistance [20]. In addition, although there have been achievements in the research and development of new filter materials, the corresponding preparation processes must also be improved, and it is difficult to widely promote new materials due to their relatively high cost and difficulty of preparation [21]. Therefore, it remains necessary to reuse existing fiber filtering materials or find suitable long-term solutions.

Much research focuses on the air filter materials’ regeneration performance. Air filter regeneration methods currently include water cleaning, pulse, rapping, back blowing, and ultrasound [22,23,24,25]. Among them, water cleaning is the most widely used because it is convenient, easy, and inexpensive. The cost of treating the used aqueous cleaning solution is relatively low [22]. However, pulse, rapping, back blowing, and other methods are mainly used for industrial filter materials regeneration, with associated drawbacks including the high cost and manufacturing difficulty [23,24]. In recent years, ultrasonic cleaning technology has gradually been applied to cleaning air filter materials due to its wavelength, energy concentration, and strong penetration [25]. However, ultrasonic cleaning is difficult to promote on a large scale, as it is technically difficult and requires a high investment.

Additionally, related research focuses on the development of new regenerative filter materials [26,27,28]. Graphene and its derivatives have become a hot topic in materials synthesis due to their specific characteristics [26,27]. Compared with general air filter materials, the new reduced graphite oxide air filter material has increased fiber strength, such that the wrapping layer does not easily fall off; it has strong stability and can withstand greater external forces [26,27]. Notably, it also has the prerequisite capacity for multiple cleanings and utilizations, and cleaning effectively removes impurities such as particles attached to its fibers and adhering to the inside of the fibers [27]. However, there is little research regarding the regeneration performance of the new reduced graphite oxide air filter material, and even less research regarding the parameters for water cleaning it and its performance stability post cleaning.

Therefore, to solve the practical problems noted above, this study assessed the regeneration performance of reduced graphite oxide filter material using water cleaning, and analyzed the relevant parameters. It provides a base-line reference for the regeneration and utilization of air filter fiber under the dual-carbon target.

## 2. Methods

### 2.1. Performance Parameters

The cleanliness of air filter materials after cleaning was used as the evaluation standard for the amount of dust in air filter materials after cleaning, as calculated using Equation (1) [29].

(1) $$\eta_{1}=(1-\frac{m}{M})\times 100\%$$

where *η*_1_ is cleanliness (%); *m* is the mass of residual dust in the filter material after cleaning (g); and *M* is the initial mass of the clean filter material (g).

The air filters’ filtration efficiency was calculated using Equation (2) [8,19].

(2) $$\eta =\frac{C_{1}-C_{2}}{C_{1}}\times 100\%$$

where *η* is the filtration efficiency of air filters (%); *C*_1_ is the concentration of particulate matter before filtration (μg/m^3^); and *C*_2_ is the concentration of particulate matter after filtration (μg/m^3^).

The air filters’ filtration resistance was calculated using Equation (3) [8,19].

(3) $$\Delta P=P_{2}-P_{1}$$

where *P*_1_ is the static pressure before filtration (Pa); and *P*_2_ is the static pressure after filtration (Pa).

### 2.2. Experimental Systems

A MTQ300 g electronic analytical balance was used for weight measurement, and it was supplied by Shenzhen Mobil Electronics Co., Ltd., China; the measuring range was 0.03~300 g, and the measurement accuracy was 0.001 g. An XCS-101-0BS electrical blast drying oven was used for drying, and it was supplied by Shaoxing shi Shang Cheng Instr ument equipment Co., Ltd., China; the temperature ranged from room temperature to 300 °C. A JSM-6510LV scanning electron microscope was used for analysis, and it was supplied by Japan Electronics Co., Ltd., Tokyo, Japan; its magnification was 5~30 million times, and its resolution was up to 3.0 nm. An HD37AB1347 Indoor Air Quality Monitor was used to measure velocity, and it was supplied by DeltaOHM Co., Ltd., Padova, Italy; with an accuracy range of ±3%. An HD2114P.0 Portable Micromanometer was used to measure filtration resistance, and it was supplied by DeltaOHM Co., Ltd., Italy; with an accuracy of ±(2% reading + 0.1 m/s). The difference pressure range was ±0.4% F.S. A TSI7525 Indoor Air Quality Meter Measuring Instrument was used to measure temperature and humidity, and it was supplied by TSI Instrument Beijing Co., Ltd., China, with a measurement range of 0~60 °C, a measurement accuracy of ±0.6 °C, and 0.1 °C resolution. The relative humidity measurement range was 5~95% RH, measurement accuracy was ±3% RH, and the resolution was 0.1% RH. A GRIMM1.109 Portable Aerosol Spectrometer was used to measure concentrated particles before and after air filters were applied, and it was supplied by Beijing Saak-Mar Environmental Instrument Ltd., China. The upper limit of the concentration that could be counted was 2,000,000 P/L. Particles ranging from 0.25 to 32 μm in diameter could be separated into 31 channels. The repeatability was 5%. The average concentrations over 5 min before and after testing air filter materials were recorded [8]. Data were analyzed using mean values to reduce experimental error. The experimental setup was developed according to China’s national standard [30]. Distributing measuring points were applied according to China’s national standard [31].

The relevant literature and experimental data indicate that water temperature has a relatively low impact on the water cleaning effect in practical engineering [8,32]. Therefore, this study only considered the effects of washing intensity and washing time on the water cleaning effect. Under the condition of controlling a single variable, the designed washing intensities were 8 L/(s·m^2^), 14 L/(s·m^2^), and 20 L/(s·m^2^), in sequence. The washing times were 10 s, 20 s, 30 s, 40 s, and 50 s. The clean filter material was weighed separately; the dust was collected, and maintaining the water flow could completely clean the entire structure of the material. Next, it was cleaned before weighing, and then Equation (1) was applied.

## 3. Results and Discussion

### 3.1. Determination of Water Cleaning Parameters

**Figure 1:**
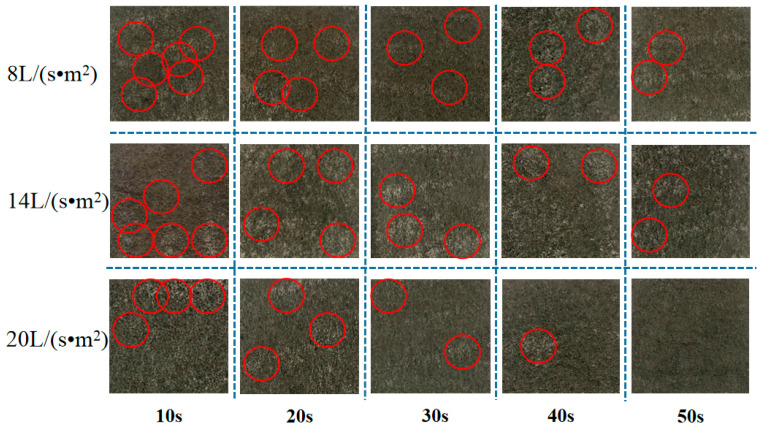
Water cleaning process.

**Figure 2:**
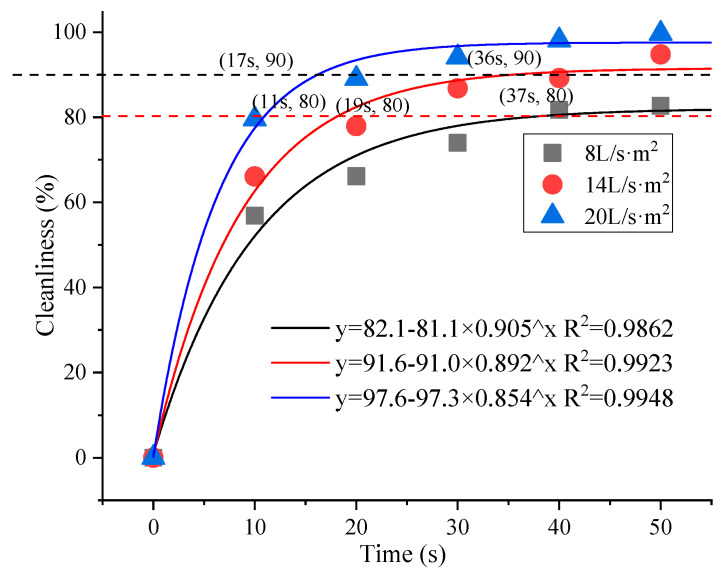
Correlation factors analysis.

Cleanliness using the different washing intensities and times was assessed under the condition of ensuring control of a single variable change. The specific operation and physical changes are shown in Figure 1, and the analysis of the related factors is shown in Figure 2.

It can be seen from these figures that water cleaning at different water flow velocities had different cleaning effects on the reduced graphite oxide air filter materials. Washing intensity and time both impacted the cleaning effect. It can be verified using the physical maps in Figure 1 that sections of dust might appear on the surfaces and fiber interior of the reduced graphite oxide air filter materials under different water cleaning conditions. When post-washing cleanliness reached 80% of the original cleanliness, the corresponding times for 8 L/(s·m^2^), 14 L/(s·m^2^), and 20 L/(s·m^2^) were 37 s, 19 s, and 11 s, respectively. Only washing with water flow velocities of 14 L/(s·m^2^) and 20 L/(s·m^2^) could achieve post-washing cleanliness equal to 90% of the original cleanliness (with corresponding wash times of 36 s and 17 s, respectively); this could not be achieved using a water flow velocity of 8 L/(s·m^2^). Therefore, the experiment used 90% cleanliness of the original filter material, taking into account the water consumption and economic cost. A 20 L/(s·m^2^) water flow velocity and 17 s cleaning time were selected as the optimal operating parameters for multiple water washes and testing the regeneration performance.

### 3.2. The Change in Filtration Efficiency with Filtration Velocity and Cleaning Times

**Figure 3:**
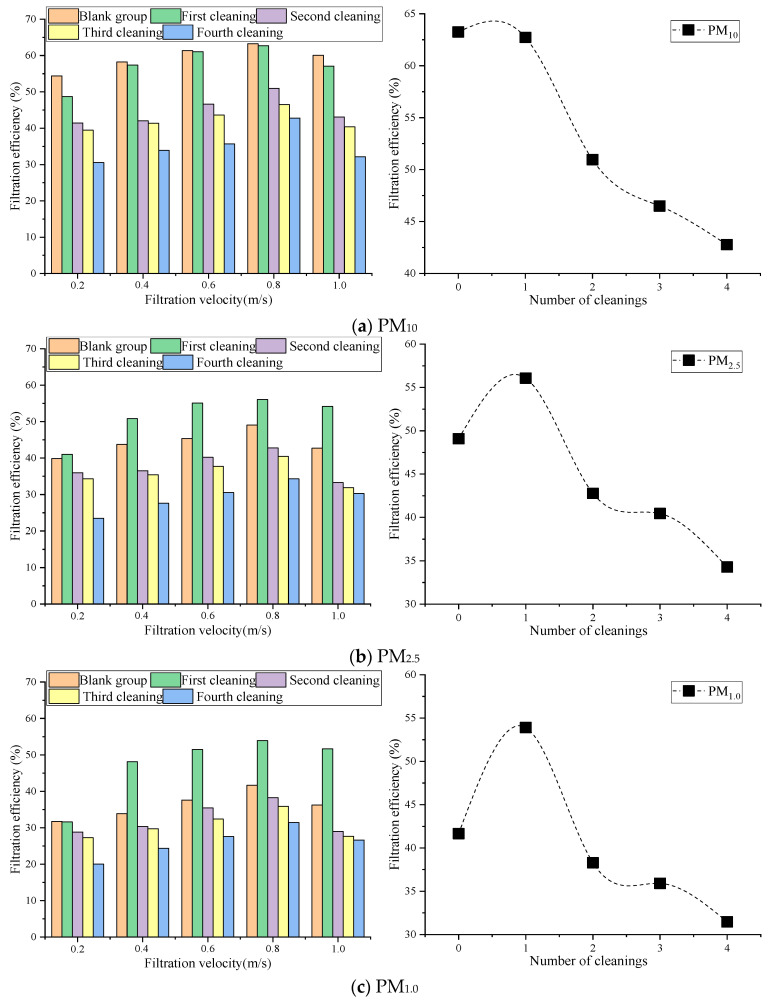
Impact of cleaning times on filtration efficiency.

Dust collected from the air conditioning systems was used for the dust charging experiments. Levels of particulate matter (PM) with aerodynamic diameters less than 10 μm (PM_10_), 2.5 μm (PM_2.5_), and 1.0 μm (PM_1.0_) were assessed using the water cleaning process. The trend of filtration efficiency with filtration velocity and four cleanings using the optimal water cleaning parameters are shown in Figure 3.

Figure 3 shows that the filtration efficiency first increased and then decreased with increases in the filtration velocity of the optimal water cleaning parameters. When the filtration velocity was 0.8 m/s, the filtration efficiency reached its maximum. Overall, the fiber’s PM_10_ filtration efficiency gradually decreased as the number of cleanings increased; the difference between the first cleaning and the original efficiency was not significant. The fiber’s PM_2.5_ and PM_1.0_ filtration efficiencies showed a different trend during the first cleaning. With an increase in cleaning times, PM_2.5_ and PM_1.0_ first increased and then decreased. The main reason for this was that the synthetic reduced graphite oxide air filter material was similar to a membrane-covered filter material in the process of dust charging cleaning [33]; the reduced graphite oxide completely wrapped the fibers, which held the dust on the fiber surfaces to achieve surface filtration [34]. After water cleaning, large particles were directly washed away, and some small particles entered the fibers’ interiors. This caused the gaps between the fibers to become smaller, which led to less significant changes in the large particles, but obvious changes in the small particles. As the number of cleanings increased, the small particles inside the fibers gradually reached the edge of the fiber and eventually washed out. However, this caused the size of the gaps between the fibers to increase, which reduced their interception effect and led to a gradual decrease in the filtration efficiency [35].

Compared to the blank group, the fiber’s PM_10_ filtration efficiency decreased by 0.8%, 19.4%, 26.5%, and 32.4% after the first to fourth cleanings, respectively. The fiber’s PM_2.5_ filtration efficiency increased by 12.5% after the first cleaning, and decreased by 12.9%, 17.6%, and 30.2% after the second to fourth cleanings, respectively. The fiber’s PM_1.0_ filtration efficiency increased by 22.7% after the first cleaning, and decreased by 8.1%, 13.8%, and 24.5% after the second to fourth cleanings, respectively. The relevant standard indicates that the air filter’s filtration efficiency after water cleaning should not be lower than 85% of its filtration efficiency before cleaning [30]. For example, the fiber’s PM_2.5_ filtration efficiency decreased by 12.9% after the second cleaning, and by 17.6% after the third cleaning. Therefore, the reduced graphite oxide air filter materials could be water washed twice under the condition that the cleanliness reaches 90% of the original filter material’s cleanliness. It could not reach the standard’s 85% pre-washing cleanliness level after more than two cleanings [30].

### 3.3. The Change in Counting Filtration Efficiency with Filtration Velocity and Number of Cleanings

**Figure 4:**
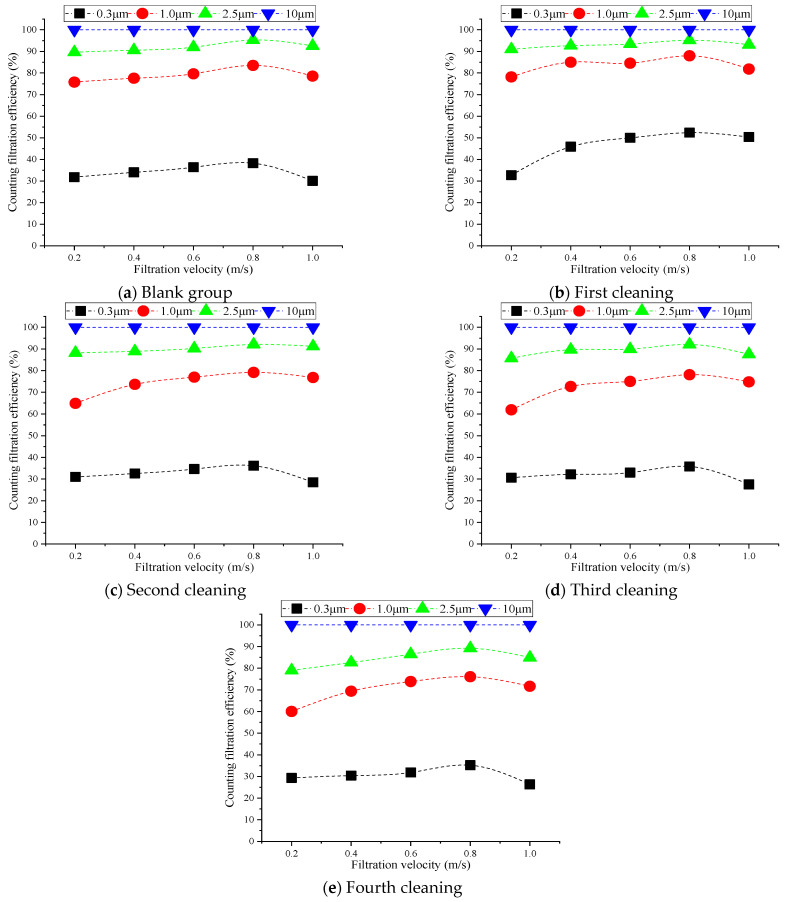
Impact of number of cleanings on counting filtration efficiency.

Figure 4 illustrates the trends of counting the filtration efficiency with filtration velocity and the number of cleanings under the same conditions mentioned above.

Figure 4 shows that the counting filtration efficiency first increased and then decreased with the increases in filtration velocity. The change in the small particles was relatively obvious, whereas the difference in the large particles was not significant. This was because the outermost large particles on the fiber surface fell due to the impact of the water flow during the cleaning process, and a portion of the particles in direct contact with the fiber surface was washed away by the water flow. Some particles entered the fibers’ interiors due to the uneven distribution of the water washing action points, which caused them to adhere to the fiber structures instead of being cleaned off. This reduced the porosity between the fibers and made the fiber structure denser [35]. However, the small particles were affected by Brownian motion and diffused in the fibers. At this stage, particles were more easily trapped by the combined effects of diffusion and interception. The counting filtration efficiency was the highest after the first cleaning; however, as the number of cleanings increased, the counting filtration efficiency of the small particles showed a downward trend. This was because the particles inside the fiber were gradually cleaned. Particles attached to the fibers fell off during multiple cleanings and were washed away from the fiber, which impacted the fiber structures; as a result, the counting filtration efficiency gradually decreased. 

It is common to observe cleaned filters with the naked eye in practical engineering. When a section of a filter’s structure is found to be damaged after cleaning, it is usually directly replaced. This is a disadvantage when cleaning using water. Unstable water pressure and uneven water flow can easily lead to fiber damage, and can also affect the filtration efficiency after cleaning [36]. Water cleaning primarily affected the filtration efficiency of particulates sized 0.3–2.5 μm. As the number of cleanings increased, the counting filtration efficiency gradually decreased.

### 3.4. The Change in Filtration Resistance with Filtration Velocity and Number of Cleanings

**Figure 5:**
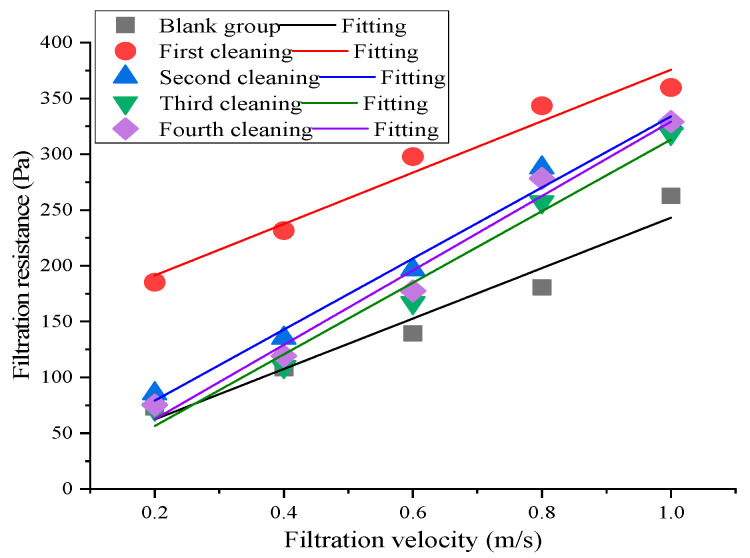
Impact of number of cleanings on filtration resistance.

Figure 5 illustrates the trends of filtration resistance with filtration velocity and number of cleanings under the same conditions mentioned above.

Figure 5 shows the differences in the number of cleanings’ impact between the resistance ranges before and after water washing within the filtration velocity range. The filtration resistance of the reduced graphite oxide air filter material after water washing slightly increased, and the resistance change after the first cleaning was the largest. This was primarily because although the vast majority of dust particles on the filter materials were impacted by the action of water flow under the same conditions, some particles entered the fibers’ interiors due to water flow uniformity issues. As a result, the filter materials could not be thoroughly cleaned. In addition, the reduced graphite oxide air filter materials are similar to surface filters [34]. Some particles might have entered the fibers, decreasing the filter material’s porosity; thus, affecting the air flow velocity’s uniformity, which increased the filtration resistance. As the number of cleanings increased, the particles that had entered the filter fibers’ interiors might have been directly washed out by the water flow action, or directly dropped; the filtration resistance declined, but remained higher than the original resistance range. This was because it was difficult to completely remove the particulates from inside the fibers during the water cleaning. In addition, impurities, such as particulates, may have bonded with the fibers due to prolonged non-cleaning, which made them more difficult to remove. The filtration resistance ranges after cleaning all met the standard requirements that resistance after cleaning should not be higher than 150% of the resistance before cleaning [30]. 

### 3.5. Microstructure of Reduced Graphite Oxide Air Filter Material

**Figure 6:**
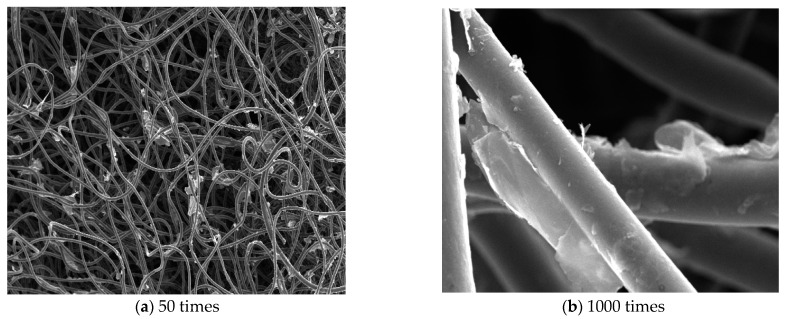
Electron microscope scanning of reduced graphite oxide air filter material.

SEM images of the reduced graphite oxide air filter material with 50 and 1000 magnification are shown in Figure 6.

As can be seen in Figure 6, the surfaces of the new reduced graphite oxide filter material had a reduced graphite oxide coating. The surfaces between the new reduced graphite oxide air filter fibers appeared relatively rough under 50 times magnification, and reduced graphite oxide was deposited on some of the fibers’ surfaces. This phenomenon was also observed in experiments described in the relevant literature [37], which verified this study’s results. Under 1000 times magnification, it could be clearly seen that some sections of the reduced graphite oxide were cross-linked between the surfaces, and also deposited in the fiber joints, which increased the filter material’s viscosity and firmness. Therefore, it has some water washing resistance, which lays the foundation for repeated cleaning and reuse. The new reduced graphite oxide air filter material has obvious advantages and good market application value considering the labor cost, operational difficulty, and material performance parameters. In situations such as underground mine environments [38,39], it will have a wide range of applications and scenarios.

## 4. Conclusions

The regeneration performance of reduced graphite oxide filter materials was studied in depth using water cleaning. The relevant parameters and filtration efficiencies after different numbers of cleanings were compared and analyzed. The conclusions are as follows:

1. The water cleaning effect was optimal when using a 20 L/(s·m^2^) water flow velocity and a 17 s cleaning time to achieve cleanliness equal to 90% of the original filter material’s cleanliness.
2. The filter’s PM_10_ filtration efficiency gradually decreased with the number of cleanings, whereas its PM_2.5_ and PM_1.0_ filtration efficiencies first increased and then decreased as the number of cleanings increased. The reduced graphite oxide completely wrapped the fibers, which held the dust on the fibers’ surfaces to achieve the surface filtration of small particles.
3. The counting filtration efficiency first increased and then decreased with the change in the filtration velocity. As the number of cleanings increased, the small particles’ counting filtration efficiency showed a downward trend. Water cleaning mainly affected the filtration efficiency of 0.3–2.5 μm-sized particulates.
4. The reduced graphite oxide air filter materials could be cleaned twice by water washing under the condition that the cleanliness reached 90% of the original filter material’s cleanliness. It could not reach the standard value of 85% before cleaning after more than two cleanings. These data are useful reference values for the evaluation of the filter materials’ regeneration performance.

## Data Availability

The study did not report any data.
